# Bridging the Divide: The Role of Motivation and Self-Regulation in Explaining the Judgment-Action Gap Related to Academic Dishonesty

**DOI:** 10.3389/fpsyg.2018.00246

**Published:** 2018-03-01

**Authors:** Jason M. Stephens

**Affiliations:** Faculty of Education and Social Work, School of Learning Development and Professional Practice, University of Auckland, Auckland, New Zealand

**Keywords:** moral judgments, self-regulation, moral disengagement, academic dishonesty, high school students

## Abstract

There is often a divide between moral judgment and moral action; between what we believe we ought to do (or not do) and what we do. Knowledge of this divide is not new, and numerous theories have attempted to offer more robust accounts of ethical decision-making and moral functioning. Knowledge of widespread academic dishonesty among students is also not new, and several studies have revealed that many students report cheating despite believing it is wrong. The present study, involving cross-sectional survey data from a sample of secondary students (*N* = 380) in the United States, contributes to the literature on this important area of theory and research by fulfilling three broad purposes. The first purpose concerned the assessment of students' judgments related to academic dishonesty, and offered evidence for the utility of a new instrument that measures what domain (personal, conventional, or moral) students use to categorize various types of cheating behavior rather than how much they believe it to be wrong. The second purpose involved exploring the relations between domain judgments and engagement in academic dishonesty, and results provided evidence for the hypothesis that students who believed an action to be morally wrong would be less likely to report doing it. Finally, the third and most important purpose of the study involved bridging the divide between moral judgment and action of academic dishonesty by testing competing theoretical models of moral functioning. Results indicated that the data demonstrated the best fit to a modified version of the hypothesized four-component model, whereby self-regulation (in the form of selective moral disengagement) played a significant mediating role in the relations between moral judgment and academic dishonesty, and that moral judgment also affected self-regulation indirectly through moral motivation (i.e., responsibility judgments). In brief, findings from this study offer support for the contention that moral functioning is both multi-component and effortful. Moral judgment is important, but only one of several components needed for effective moral functioning, and motivation and self-regulation play critical mediating roles in helping to bridge the divide between judgment and action.

## Introduction

He who knows what good is will do good.

-Socrates (in Gaarder, 1996)

One need not be a philosopher or psychologist to understand that moral judgment is not a guarantee of moral action. There is often a divide between that which we say we ought do—or not do—and that which we do. Many students, for example, cheat, even when they believe it is wrong to do so (e.g., Anderman et al., 1998; Jordan, 2001; Stephens and Nicholson, 2008). This divide between belief and behavior has been rich theoretical ground among philosophers for millennia and psychologists of the past century. Among the former it has been called the “Thought/Action problem” (Locke, 1983) and the “judgment-action gap” among the latter (Blasi, 1980). In either case, the problem is one of explaining the oft-observed insufficiency of moral judgment to produce moral action; and the questions to be answered concern identifying and explaining the psychological, social, and/or situational factors that either exacerbate or help bridge the divide.

Toward this end, numerous theories of ethical decision-making and moral functioning have been proffered (e.g., Sykes and Matza, 1957; Festinger, 1962; Fishbein and Ajzen, 1972; Blasi, 1980, 1983, 1984; Kohlberg and Candee, 1984; Bandura, 1986; Rest, 1986; Beck and Ajzen, 1991; Weiner, 1995; Rest et al., 1999; Bergman, 2002; Roberts et al., 2017). Albeit operationalized with different constructs, motivation and self-regulation are central to all theories in explaining or bridging the gap between moral cognition and action. The present study explores the power of some of the most prominent models in explaining the epidemic of academic dishonesty among adolescents. More precisely, the present paper tests the extent to which *responsibility judgments* (Kohlberg and Candee, 1984) or *moral obligation* (Beck and Ajzen, 1991) and *mechanisms of moral disengagement* (Bandura, 1986, 1990, 1999) or *techniques of neutralization* (Sykes and Matza, 1957) mediate the relationship between high school students' *moral judgment* and action related to *academic dishonesty*.

In the field of moral psychology, Rest's (Rest, 1986; Rest et al., 1999) Four-Component Model of (FCM) of moral functioning is among the most widely known. As a student of Lawrence Kohlberg, and his cognitive-structural theory of moral development (Kohlberg, 1969, 1984), Rest was familiar with the judgment-action gap and developed the FCM to address it. The FCM postulates that effective moral functioning involves the integrated use of four processes: sensitivity, judgment, motivation, and action. Essentially, when immersed in a situation involving moral values or principles, one must be not only aware of the moral values or principles at stake, and render a judgment (ideally rooted in principled reasoning) concerning the what one should do, we must also—if the judgment rendered is to be enacted—be moved to act, and to regulate our thoughts, emotions, and movements in pursuit of the chosen course. Moreover, we often must overcome temptations, competing goals or desires, and occasionally barriers and obstacles, that would have us desist or follow a different (less righteous) path.

Two theoretical suppositions of the FCM are important to the present investigation. First, and most plainly, effective moral or ethical functioning involves many processes or components, each with its own purposes and skills (perceptual, cognitive, affective, and behavioral). Judgment is but one of them, and, by itself, no guarantee of moral action; necessary, but insufficient. Second, effective moral or ethical functioning is effortful, even if not always consciously so. It requires motivation and self-regulation to move our moral judgment to moral action: We must not only know the good, we must also be moved to act and exercise the self-control and ego-strength needed to do the good (or refrain from doing the bad). The latter supposition, morality as effortful, is well described in theory of deliberate honesty (Bereby-Meyer and Shalvi, 2015). Adopting a dual process model (see Kahneman, 2011) to explain dishonesty, Bereby-Meyer and Shalvi posit that “when lying serves self-interest, that is, when lying is tempting, honesty may require deliberation” (p. 195). In brief, in the face of temptation to gain personal advantage, system 2 (deliberative—slow with high cognitive load) processes are required to override system 1 (intuitive—fast and automatic) processes; that honesty may not be our “default response” and that being so requires self-control and ego-strength.

Numerous empirical studies have lent support to both suppositions. With respect to the multi-component nature of moral functioning, several studies have shown that other psychological processes (beyond reasoning and judgment) are implicated in academic dishonesty (Malinowski and Smith, 1985; Beck and Ajzen, 1991; Diekhoff et al., 1996; Jordan, 2001; Hunt and Vitell, 2006; Moores and Chang, 2006; Anderman and Murdock, 2007; Detert et al., 2008; Murdock et al., 2008; Vohs and Schooler, 2008; Shu et al., 2011; Olafson et al., 2012; Fosgaard et al., 2013; Motro et al., 2016). For example, in their widely-cited study using the theory of planned behavior, Beck and Ajzen (1991) showed that motivational factors (i.e., perceived moral obligation) and self-regulatory beliefs (i.e., perceived behavioral control) explained significant additional variance (beyond attitudes) in predicting both intentions to cheat and engagement in cheating.

In addition to psychological research, scores of studies from the field of neuroscience have also provided evidence for the multi-component and effortful nature of moral functioning (for meta-analyses, see Sevinc and Spreng, 2014; Eres et al., 2017; Han, 2017). For example, in their a meta-analysis of 40 fMRI studies, Sevinc and Spreng (2014) found that nearly a dozen regions of the brain are activated during both “active moral judgment tasks” (i.e., tasks requiring participants to render and declare a judgment) and “passive tasks” (i.e., tasks where no deliberation or explicit judgment was required, only the viewing a stimulus that contained either morally-laden or neutral content). However, while both types of tasks call on many of the same brain regions, compared to passive tasks, engagement in active moral judgment tasks demonstrated more reliable activity in the superior and anterior regions of temporoparietal junction (TPJ), angular gyrus, temporal pole, and left medial prefrontal cortex (MPFC). Whereas compared to active tasks, passive tasks more reliably activated the lingual gyrus, left amygdala and right MPFC. In short, though all moral tasks require activation in several brain regions, more demanding tasks require greater use of regions associated with deliberative, effortful thinking (System 2) and passive tasks greater use of automatic, unconscious processing (System 1) (e.g., Greene et al., 2001; Greene and Haidt, 2002).

These neuroscience studies offer an important reminder to psychologists and other researchers studying moral functioning, particularly as it relates to academic dishonesty. That is, while neuroscientists tend to elicit response (location and intensity of brain activity) by presenting subjects (at full attention enveloped in an fMRI machine) with stimuli (verbal and/or visual) that are unambiguously moral (e.g., the classic trolley dilemma involving life-and-death decisions), the latter tend to elicit response (latent attitudes as expressed by ticks on a Likert-type scale) by presenting participants (in classrooms or online) with stimuli (descriptions of behaviors) that may be ambiguous with respect to their morality (e.g., participants may not perceive or believe the behavior in question to be a moral one). The last methodological difference is the most important one. If you're trying assess ethical decision-making or moral functioning, participants must see and process that selected stimuli as morally-relevant; else you're not activating the regions of the brain associated with moral decision-making (e.g., Greene et al., 2001; Greene and Haidt, 2002).

On this point, much of the literature to date on students' beliefs and judgments about academic dishonesty hasn't been unambiguous. Most studies, that is, have assessed students' attitudes toward cheating behaviors with Likert-type scales that measure *how much* students believe academic dishonesty to be “wrong,” “serious,” or “unacceptable” (e.g., Anderman et al., 1998; Jordan, 2001; Stephens et al., 2007). While these studies have produced clear and consistent results demonstrating significant negative associations between students' attitudes toward cheating and their engagement in it, they do not ask *what domain* of behavior students' might use to classify or judge academic dishonesty. In short, it's clear students see cheating as “wrong” (to varying degrees depending on the type of behavior; see, e.g., Stephens et al., 2007), but left unclear whether students believe the behaviors to be “morally wrong” (for exceptions, see Murdock et al., 2004; McDonald et al., 2014).

In one of the few exceptions, McDonald et al. (2014) adopt social domain theory (SDT) to investigate how adolescent peers discuss social dilemmas, including one (among six) on academic dishonesty (i.e., looking at answers to a test left on the teacher's desk). SDT (Turiel, 1983; Nucci, 2001; Smetana, 2006) posits that when making social judgments, individuals consider information and issues from three distinct (with some intersecting and overlapping) domains of social knowledge: (1) *personal*, which includes those choices or behaviors regarded as matters of individual autonomy and choice, including those rooted in pragmatic or prudential concerns; (2) *conventional*, comprising cultural customs and societal rules that prescribe (or proscribe) a set of norms governing individual behavior; and (3) *moral*, actions that involve issues of justice, fairness, welfare, and rights—actions that one should or should not do, even in the absence of conventions. McDonald et al. (2014) found that while adolescents mention moral reasons more frequently than conventional or personal issues when discussing issues such as a telling on a friend or spanking a child, they were more likely to discuss conventional and pragmatic/prudential concerns when talking about academic dishonesty (or one form of it, and a rather risky one). In short, more research is needed that makes these important domain distinctions when investigating how students perceive, reason through, and judge the myriad types behaviors associated with academic dishonesty (from plagiarizing a few sentences to using unpermitted notes during an exam).

With this mind, the present study involved the use a new instrument designed to assess students' domain judgments of several types of academic dishonesty; one rooted in SDT and guided by others (Kuther and Higgins-D'Alessandro, 2000). Instrument development and validation, however, was not the primary goal of the present study; but necessary to fulfill it. As noted above, primary purpose was to test competing theoretical models of moral functioning that might offer some purchase on understanding the judgment-action gap as it relates to academic dishonesty. Of most relevance to the present study were motivational and self-regulatory variables—*responsibility judgments* and *moral disengagement—*that might directly or indirectly affect the relations between judgment and action. As summarized next, both constructs have strong theoretical and empirical basis for being part of the hypothesized four-component model of moral functioning being tested as the benchmark in this study.

As theorized by Kohlberg (Kohlberg, 1984; Kohlberg and Candee, 1984), *responsibility judgments* are “second-order” judgments concerning one's personal moral duty to act in accord with one's “first-order” *deontic choices* (i.e., judgments concerning the right or just course of action to pursue in the face of a moral dilemma). These responsibility judgments produce a sense of personal accountability to “follow through” and “perform the right action” (Kohlberg and Candee, 1984, p. 57). Using related terminology, other theorists have also written about the importance of *moral obligation* (Beck and Ajzen, 1991) or *judgments of responsibility* (Weiner, 1995) in motivating and regulating moral behavior, and empirical research has consistently shown negative associations between these second-order judgments and academic dishonesty (e.g., Beck and Ajzen, 1991; Carpenter et al., 2004; Harding et al., 2007; Stephens et al., 2007; Murdock et al., 2008; Alleyne and Phillips, 2011). In the present study, responsibility judgments are posited to mediate the relations between first-order judgments and cheating behavior.

Kohlberg and Candee (1984) also recognized that *ego controls* and other non-moral skills were needed for moral judgment (deontic and responsibility) to become moral action. As Blasi (1983) described it, “Since a judgment of responsibility concerns the necessary relation between agent and action, not to act according to one's judgment should be perceived as a substantial inconsistency, as a fracture within the very core of the self, *unless neutralizing devices are put into operation*” (p. 201; emphasis added). These “neutralizing devices” have a rich theoretical history in psychology, as *ego defense mechanisms* (Freud, 1936/1993; Freud and Strachey, 1989), and in sociology, as *techniques of neutralization* (Sykes and Matza, 1957). More recently, Bandura (1986, 1990, 1999) has called them *mechanisms of moral disengagement*. Despite the myriad labels, the psychological purpose is the same: to protect one from self-recrimination for immoral (in)action by obscuring or denying one's personal responsibility for (in)action.

As further described in Bandura's (1991) social cognitive theory of self-regulation, human behavior is a complex product of mechanisms and subfunctions. The latter include the monitoring of one's behavior, judging it in relation to personal standards and those of others, and affective self-reactions to that conduct. When activated these self-regulatory subfunctions work together to produce behavior consistent with judgments and the standards undergirding them. However, Bandura (1986, 1999) further argued that moral self-regulation can be selectively deactivated, and proposed a set of eight *mechanisms of moral disengagement* that enable this deactivation process (e.g., euphemistic labeling, advantageous comparison, disregarding or distorting the consequences, displacement of responsibility, diffusion of responsibility, attribution of blame, dehumanization, and moral justification).

Empirical research has demonstrated a strong positive associations between moral disengagement (or *neutralization*, as the construct has been operationalized in some studies) and academic dishonesty: as former increases, so too does the latter (e.g., Haines et al., 1986; LaBeff et al., 1990; McCabe, 1992; Diekhoff et al., 1996; Cava, 2000; Stephens and Gehlbach, 2007; Stephens et al., 2007; Zito and Mcquillan, 2010; Farnese et al., 2011; Shu et al., 2011; Gabbiadini et al., 2013). McCabe (1992), for example, found that displacement of responsibility was the most prevalent mechanism employed by undergraduates: 61% of those that reported cheating rationalized their behavior by blaming others and/or some aspect of the situational context. Similarly, Evans and Craig (1990) found that displacement of responsibility to the teacher was most common among college-bound and high-achieving high school students.

### Purposes, questions, and hypotheses

With the foregoing theory and research in mind, the present study has three distinct purposes. As presented below each purpose is framed by a set of research questions and hypotheses.

The first purpose concerns students' judgments related to academic dishonesty. Specifically, the present study employed the use of a new instrument rooted in social domain theory (SDT; Turiel, 1983; Nucci, 2001; Smetana, 2006) to assess students' judgments related to a range of cheating behaviors. As reviewed above, much of the research to date has used attitudinal scales to assess *how much* some action is wrong and not *what domain* of action it belongs. With this important distinction in mind, the following research questions (RQ) and hypotheses (H) were formulated:

RQ1: *What are students' domain judgments (i.e., personal, conventional, or moral) related to academic dishonesty?*
  H1: Previous research using SDT (McDonald et al., 2014) it was hypothesized that the majority of students would judge academic dishonesty to be a matter of convention (i.e., wrong because school rules proscribe the behavior), with fewer students judging the behaviors assessed as morally wrong, and fewer still as a personal choice.
RQ2: *To what extent do these domain judgements vary based on the type of academic dishonesty involved (i.e., assignment cheating, plagiarism, and test or exam cheating)?*
  H2: The overall distribution pattern described in H1 was expected to vary significantly by the type of academic dishonesty in questions. Specifically, it was hypothesized that students would be more likely to judge assignment cheating as personal choices or conventional issues, and more likely categorize test cheating as morally wrong. This hypothesis is based on previous findings that have consistently shown variation in students' rating of these of these types of academic dishonesty (e.g., Franklyn-Stokes and Newstead, 1995; Stephens and Gehlbach, 2007; Stephens et al., 2010)

The second purpose of the present study was to explore the associations between participants' domain judgments and actions related to academic dishonesty. In using SDT (e.g., Turiel, 1983; Nucci, 2001; Smetana, 2006) to operationalize and assess students' judgments related to academic dishonesty, the present investigation takes seriously the idea that judgments may vary not only in degree (as typically measured) but also in kind (i.e., as personal, conventional, or moral issues), and that the latter judgment is an important determinate of behavior.

RQ3: *To what extent are domain judgments of academic dishonesty associated with self-reported engagement in them? That is, do students who believe a behavior to be “morally wrong” less likely to report doing it?*
  H3: In keeping with the previous research described above (e.g., Anderman et al., 1998; Jordan, 2001; Murdock et al., 2004; Stephens et al., 2007), it was hypothesized that students' who judge behaviors to be morally wrong would report lower rates of engagement in that behavior compared to students who judged the behavior to be a personal choice or social convention.

**Figure 1:**
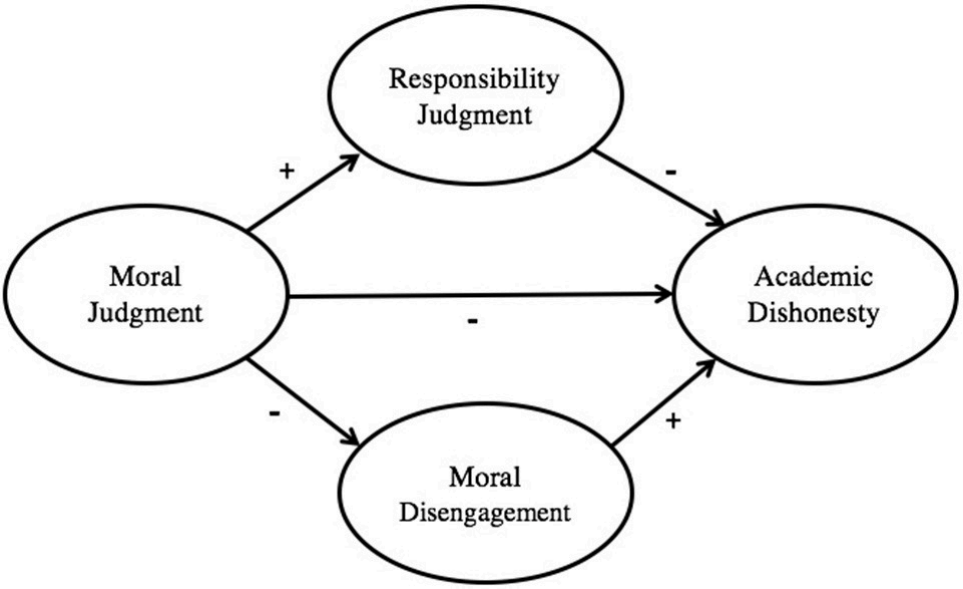
Hypothesized model of moral functioning depicting the direct and indirect relations between moral judgment and academic dishonesty.

The third and final purpose of this study was to test a multi-component model of moral functioning using responsibility judgment and moral disengagement as mediators in the relations between moral judgment and academic dishonesty (see Figure 1). More precisely, this study compares the goodness of fit of the hypothesized model (predicting multiple mediation) with the fit of other theoretical models of moral functioning. The latter models include a simple bivariate model of the direct effects of moral judgment on academic dishonesty (as Socrates would have it), a tri-variate model with responsibility judgment mediating the relations between judgment and dishonesty (consistent with Kohlberg and Candee, 1984), and another tri-variate model with moral disengagement as the mediator (as suggested by Bandura, 1986, 1990).

RQ4: Among several competing models of moral functioning, which is the most robust in predicting academic dishonesty? Specifically, does the data collected offer a better fit to hypothesized model or to those suggested by others?
  H4: As depicted in Figure 1, and consistent with previous research (Hunt and Vitell, 2006; Detert et al., 2008; Roberts et al., 2017), moral judgment was expected to have both direct and indirect associations with academic dishonesty. Specifically, moral judgment was hypothesized to have a direct negative relationship with academic dishonesty as well as indirect associations through both responsibility judgment and moral disengagement. As indicated by the notations, the former was expected to be positively associated with judgment and negatively with dishonesty, while the latter negatively associated with judgment and positively with dishonesty.
  H5: The hypothesized model was expected to demonstrate a good fit, and a significantly better one than any of three competing models (i.e., a bivariate model of the direct effects of moral judgment on academic dishonesty, and two tri-variate models, one with responsibility judgment and other with moral disengagement as mediators in the relations between judgment and dishonesty (cf. Kohlberg and Candee, 1984; Bandura, 1986, 1990).

## Methods

### Participants and procedures

This study was carried out in accordance with basic principles of the Declaration of Helsinki; namely, respect for the individual right to self-determination and the right to make informed decisions regarding participation in research, both initially and while completing the survey. Specifically, all students enrolled in the school, and their parents, were notified of study approximately 1 month before data collection toward the end of the school year. They were informed that the study would involve the completion of a short (10–15 min) survey; that the survey would ask students to report on their perceptions, beliefs and behaviors related academic integrity during the past school year; and that students' participation would be both voluntary and anonymous. Importantly, to guarantee anonymity, students were not asked to provide their names or any unique identification numbers on the survey; nor were they asked to provide traditional written consent in the form of providing a name or signature. Those interested in participating simply used the link provided (voluntarily and in their own time), and were free to withdraw their participation at any time without reason.

A total of 523 students (approximately 80% of the school population) from a highly selective private high school in the northeastern United States volunteered to participate in the study. Eighty-one of the 523 students (15.5%) who began the survey exercised their right to withdraw their participation and did not complete the survey. Another 62 students (14.0%) were removed as multivariate outliers using Mahalanobis distance values (as detailed below). Thus, the final sample included 380 students: 54.2% female; 26.5% freshmen, 26.8% sophomores, 20.7% juniors, and 26.0% seniors; 56.5% Caucasian, 18.1% Asian-American, 7.7% African-American, 5.9% Hispanic, and 11.7% multiethnic/other.

### Measures

The online survey was comprised of original and adapted measures.

#### Domain judgment

Based on the social cognitive domain theory (Turiel, 1983; Nucci, 1994; Smetana, 2006), an original measure was developed to assess students' judgments of various forms of academic dishonesty. Specifically, the new measure presented participants with a list of 15 behaviors and asked them to, “Please categorize the following behaviors into one of the three categories described below. Please do so according to YOUR opinion, values or beliefs” (emphasis in original). The three categories were presented immediately below this prompt and read: *Neither right nor wrong* (Personal Choice); *Wrong because it's against societal laws, rules or customs* (Social Violation), and *Wrong regardless of laws or rules* (Morally Wrong). The 15 behaviors included six items from Stephens et al. (2007) measure of academic dishonesty and ten original items. The latter were developed by the author and included a range of behaviors (from deciding what clothes one wears to bullying another student) designed to align with the three domains of SDT. All items were reviewed by other experts in the field before being pilot tested and revised.

The precise wording of all items is presented below in Table 1, along with results from an EFA with ML estimation and oblique minimization. As detailed, results from this analysis yielded a four-factor solution (all eigenvalues >1.00, cumulative variance = 50.98%). The first and strongest factor (accounting for 24.51% of variance; all factor loadings >0.30) included all six items related to academic dishonesty. As indicated by the distribution of coefficients in the pattern matrix, the second factor (with two items involving lying) and the third factor (with four items comprised of conventional violations such as speeding and smoking marijuana) were also conceptually clear, had item loadings >0.30, and only once instance of cross-loading. The latter involved the item about teasing or bullying, and was also the single item of the fourth factor. Three items (8, 11, and 15) were not significantly associated (loadings <0.30) with any of the four factors extracted. In short, results from this EFA indicated a single six-item factor related to academic dishonesty, and was carried forward for subsequent model testing (see Results). Given the focus of the present study, the latter three factors and their items were not considered for further analyses here.

**Table 1:** Pattern matrix from an exploratory factor analyses of domain judgments items.

**Item**	**Factor**			
	**1**	**2**	**3**	**4**
1 Copying another student's homework and submitting it as your own work.	0.597			
2. Collaborating with other students when the teacher asked you to work alone.	0.301			
3. Paraphrasing or copying a few sentences without citing the source in a paper you submitted.	0.479			
4. Using unpermitted notes or books during a test or exam.	0.739			
5. Copying from another student during a test or exam.	0.846			
6. Letting another student copy from your test or exam.	0.616			
7. Teasing, taunting or bullying someone.			0.383	−0.500
8. Deciding what clothes to wear.				
9. Lying to a parent about something significant.		0.516		
10. Driving over the speed limit.			0.553	
11. Getting a new haircut.				
12. Not paying for a parking space.			0.416	
13. Smoking marijuana.			0.331	
14. Lying to a teacher about something significant.		0.804		
15. Bending the rules to win in sports.				
Eigen value	3.68	1.43	1.39	1.15
% of variance explained	24.51	9.53	9.28	7.66

*EFA using ML estimation and oblique minimisation. Factor loadings < 0.30 are not shown*.

#### Responsibility judgment

Conceptually rooted in Kohlberg's (Kohlberg, 1984; Kohlberg and Candee, 1984) theorizing, as well as Beck and Ajzen's (1991) notion of moral obligation, an original three-item measure was developed to assess students' “responsibility judgment” related to academic integrity. Specifically, participants used a seven-point Likert-type scale (−3 = *Strongly Disagree* to +3 = *Strongly Agree*) to indicate how strongly they felt it was their responsibility to refrain from three types of academic dishonesty (i.e., “I believe that it's my responsibility NOT to cheat on homework and other school assignments,” “It is my responsibility as a student NOT to cheat on tests or exams,” and “I feel personally obligated as a student NOT to plagiarize the work of others.”

#### Moral disengagement

In order to assess students' tendency to disengage their sense of moral responsibility for refraining from cheating, a six-item scale based on Bandura et al. (1996) measure of moral disengagement was created. Specifically, students used a seven-point scale (−3 = *Strongly Disagree* and +3 = *Strongly Agree*) to indicate how strongly they felt it was not their responsibility to refrain from cheating under various circumstances (e.g., “One student should not be blamed for cheating that was started by others,” “If students have bad teachers they cannot be blamed for cheating,” and “It is alright to cheat to help your friends”).

#### Academic dishonesty

In order to assess students' engagement in academic dishonesty, we developed a six-item scale based on an existing measure by Stephens et al. (2007). Importantly, the six items were reiterations of the six items on the measure of domain judgments described above. However, in this section of the survey, they were worded in past tense and participants were asked to use a five-point scale (where 1 = *Never*, 2 = *Once or twice this year*, 3 = *About monthly*, 4 = *About weekly* and 5 = *Almost daily*) to report how often they had engaged in each of the behaviors described. Specifically, they were asked, “Since the beginning of THIS SCHOOL YEAR, how often have YOU engaged in the following behaviors?” (emphasis in original).

### Data cleaning

Before testing the five hypotheses, the data collected were subjected to several diagnostic examinations in order to “clean” them for data analyses (Van den Broeck et al., 2005). First, as noted above, 81 volunteers withdraw with their participation before completing the survey, and were removed from the sample for missing (not at random) data. Second, of the remaining 442 participants, 398 (90%) completed all items on the survey, and the other 44 (10%) missed one to three responses among the potential items (*n* = 42, including four demographic variables). Specifically, only two participants missed three responses—all demographic questions (e.g., sex, race, grade)—and only three participants missed two responses—also mostly demographic questions. The remaining 40 participants with missing data had only one response missing. Overall, among 18,564 potential data points (i.e., 442 × 42), there were 51 missing entries (0.27% missing), with the percentage of missing values for each item ranging from 0.0 to 1.1%. Missing data procedures in SPSS where used to test the assumption that responses were missing completely at random, and thus, that the data were suitable for imputation procedures using expectation maximization (Little and Rubin, 2014). A nonsignificant value for Little's test (χ^2^ = 810.91, *df* = 814, *p* = 0.524) was obtained, and all missing values were imputed.

Third, given assumption of multivariate normality for structural equation modeling (Ullman, 2006), the data were examined using Mahalanobis distance values. Sixty-two participants had values greater than 61.16, the critical value (*p* < 0.01) for the chi-square distribution with 38 degrees of freedom (i.e., the number of continuous, independent variables), and were removed from the sample as multivariate outliers. Finally, given the self-report nature of the data, recommendations regarding common method variance (CMV) were followed to test for spurious variance attributable to the method of measurement used in the survey (Podsakoff et al., 2003, 2012). Harman's (1976) single (one)-factor test returned favorable results, suggesting that CMV would not significantly bias results. Specifically, an unrotated EFA (using principal axis factoring) on all items yielded 10 factors with eigenvalues greater than 1. Importantly, the first factor accounted for 34.1% of the variance, indicating that there was not a single method factor that accounted for the majority of the variance (Podsakoff et al., 2012).

### Data analyses

As presented in the Results, data analysis began with a confirmatory factor analysis to confirm the structure and fit of the proposed four-factor measurement model. After establishing good model fit, descriptive statistics for all four factors and their inter-correlations were computed for examination and reporting. Next, in order to test the first three hypotheses of the study, the categorical data on participants' domain judgments related to six forms of academic dishonesty examined for between-item variation and then cross-tabulated with participants' engagement in those six forms academic dishonesty (which where dichotomized, where 0 = *Did not do it* and 1 = *Did it this school year*). The latter, 3 (judgment) × 2 (action), cross-tabulation used chi-square analyses to test for under- and over-representation in observed counts (in the six cells) vs. expected counts (given the marginal frequencies). Where significant differences were indicated, the adjusted standardized residuals of the cells were used identify which cells deviated significantly (beyond ± 2 standard deviations) from their expected count. Finally, structural equation modeling was used to the fourth and fifth hypothesis concerning the fit of the hypothesized model and how it compares with competing models of moral functioning. All analyses were conducted using version 23 of SPSS and its AMOS program. Several different indexes of goodness of fit were taken into consideration, including the normed chi-square (χ^2^/*df*), root mean square error of approximation (RMSEA), and Gamma hat (Gamma) as well as the comparative fit index (CFI) and the Tucker–Lewis index (TLI). Guided by suggestions provided in Hu and Bentler (1999) and Fan and Sivo (2007), acceptable model fit was defined by the following criteria: χ^2^/*df* ≤ 3.84, CFI ≥ 0.95, TLI ≥ 0.95, Gamma ≥ 0.95, and RMSEA ≤ 0.06.

## Results

Results from the confirmatory factor analysis are reported first, followed by the descriptive statistics of and bivariate correlations of the four latent factors. Subsequently, results pertaining to each of the five hypotheses are described.

### Confirmatory factor analysis of the four-factor measurement model

**Figure 2:**
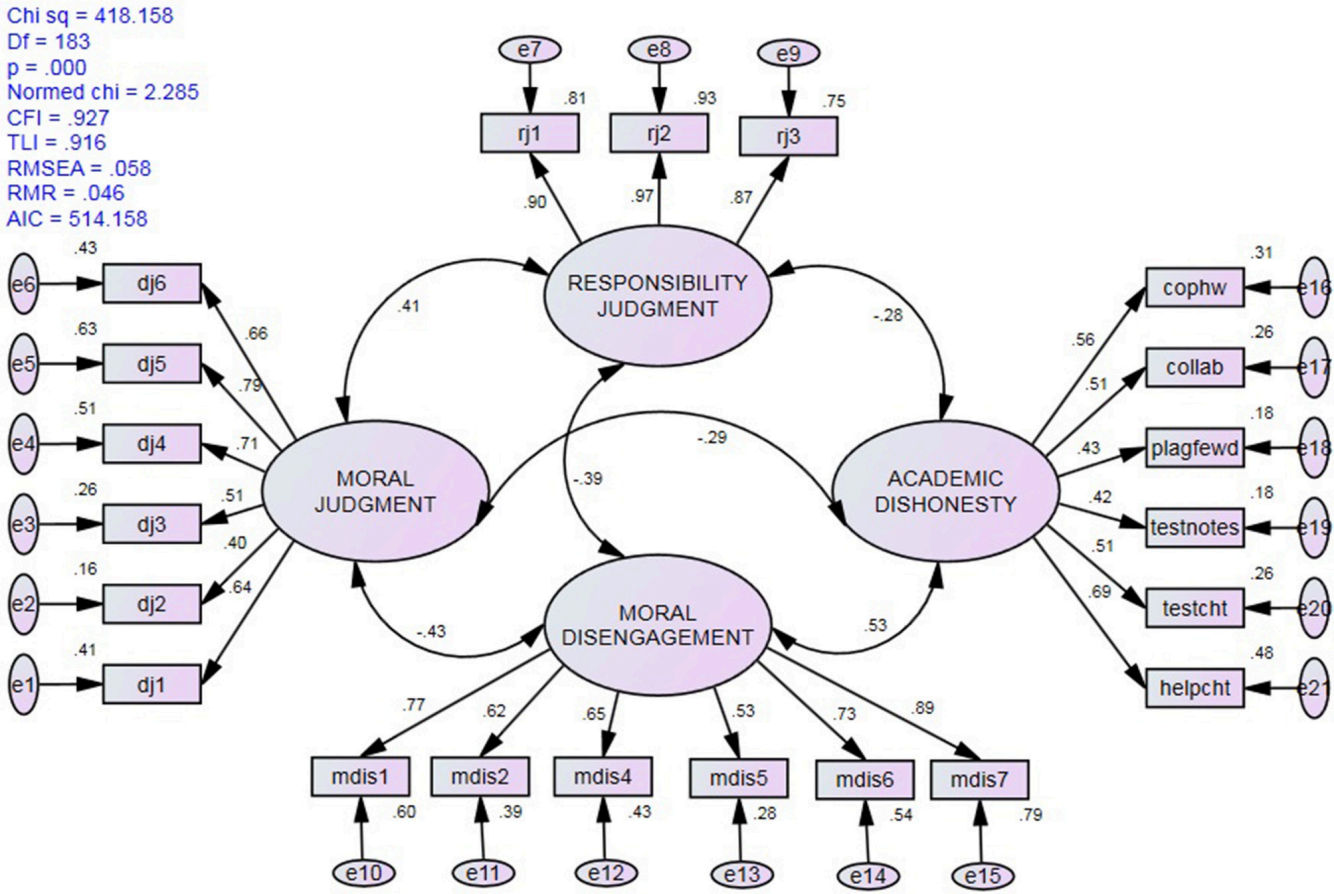
Standardized estimates and fit statistics of the four-factor measurement model.

A CFA of the four-factor measurement model produced no warnings and the data exhibited acceptable fit to it: χ^2^/*df* = 2.29, CFI = 0.927, TLI = 0.916, Gamma hat = 0.945, and RMSEA = 0.058 (90% CI = 0.051, 066). As detailed in Figure 2, all four factors were significantly inter-correlated (*r*'s = −0.28 to 0.53) and all item factor loadings were greater than 0.40 (range = 0.40–0.97). However, several items (one for moral judgment and two on academic dishonesty) had relatively low factor loadings (standardized coefficients <0.50).

### Descriptive statistics

Several descriptive statistics of the four latent factors measured in this study are detailed in Table 2. Three results are most notably. First, all factors were significantly skewed, which was not unexpected given their normative nature and social desirable responding bias that often occurs (whereby individuals over-report desired attitudes or “good” behavior and under-report controversial beliefs and “bad” behavior). Specifically, the distribution of self-reported academic dishonesty was positively skewed (skewness/standard error of skewness = 13.03), indicating that students disproportionately used the first two points of the five-point scale (i.e., *Never* and *Once or twice this year*) relative to latter end of the scales (i.e., *About weekly* and *Almost daily*). Conversely, the distribution of responsibility judgment was negatively skewed (skewness/standard error of skewness = −16.77), indicating that students disproportionately used the latter half of the seven-point scale (indicating agreement) relative to the first half (indicating disagreement). Second, Cronbach's alphas varied widely among the four factors—from a low of 0.69 for academic dishonesty and a high of 0.93 for responsibility judgment. Third and finally, the bivariate correlations were all statistically significant (*p* < 0.001) and in the directions expected (i.e., moral and responsibility judgments are negatively associated with academic dishonesty, and moral disengagement negatively related to the former and positively to the latter).

**Table 2:** Means, standard deviations, internal reliability and bivariate correlations for all four factors.

**Measure**	***M***	***SD***	**Min**	**Max**	**Skew**	**α**	**1**	**2**	**3**
1 Academic Dishonesty	1.27	0.31	1.00	3.00	13.03	0.69	–		
2 Moral Judgment	0.39	0.40	−1.00	1.00	−4.50	0.79	−0.24^**^	–	
3 Responsibility Judgment	2.49	0.78	−2.00	3.00	−16.77	0.93	−0.25^**^	0.39^**^	–
4 Moral Disengagement	−1.58	1.18	−3.00	1.83	4.75	0.85	0.37^**^	−0.39^**^	−0.37^**^

*N, 380. Min/Max, Minimum/Maximum of observed scale means; Skew, (skewness / SE of skewness)*.

** *p < 0.01*.

### Domain judgments and their relations to academic dishonesty

As detailed in Table 3, and counter to H1, a majority of participants (range = 51.1–65.8%**)** judged a majority of the behaviors (four of the six assessed) to be *morally wrong* (not *social conventions* as hypothesized). The two exceptions (i.e., unpermitted collaboration and plagiarism) were judged to be matters of *social convention* by the majority (65.1 and 61.5%, respectively). Results, however, did support the latter part of H1 as only a minority of students (range = 1.6–15.9%) judged the behaviors to be a *personal choice*. Similarly, support for H2 was also mixed. While a greater number of participants judged the three test cheating behaviors to be *morally wrong* compared to unpermitted collaboration (51.1–65.8% vs. 19.0%, respectively), an unexpectedly high number also judged the copying of homework to be *morally wrong* (59.2%).

**Table 3:** Students' self-reported judgments and actions related to academic dishonesty.

	**Judgment**			**Action**	**Action by Judgment**			
**Action**	**Personal choice**	**Social convention**	**Morally wrong**	**Did it at least once**	**Personal choice**	**Social convention**	**Morally wrong**	***χ^2^***
1 Copied Homework	4.7	36.1	59.2	31.1	44.4	35.0	27.6	3.81
2 Unpermitted Collaboration	15.9	65.1	19.0	50.0	**66.7**	49.2	*40.3*	9.85^**^
3 Plagiarized a Few Sentences	7.9	61.5	30.6	26.3	**56.7**	29.2	*12.9*	25.88^***^
4 Used Unpermitted Notes on Test	1.6	45.6	52.8	6.8	**33.3**	7.0	5.0	8.11^*^
5 Copied from Another on Test	1.6	32.6	65.8	11.3	**50.0**	12.9	9.2	10.5^**^
6 Let Another Copy from Test	10.6	38.2	51.1	15.8	**40.0**	16.6	*10.4*	22.19^***^

*N, 378–380. All numbers are percentages. Given marginal frequencies, cells with bold-faced/italicized frequencies are significantly greater/less than the expected count (adjusted standardized residual score greater than or equal to ±2.0)*.

* *p < 0.05*,

** *p < 0.01*,

*** *p < 0.001*.

Finally, results depicted in the latter four columns of Table 3 offered broad (if not complete) support for the hypothesized associations between domain judgments and academic dishonesty (H3). As indicated by the bold-faced and italicized numbers, participants who judged a behavior to be a personal choice were significantly over-represented among those that reported engagement in that behavior, while participants who judged the behavior to be morally wrong were significantly under-represented (given expected counts based on the marginal frequencies of “Action × Judgment” cross-tabulations). For example, the largest effect observed concerned plagiarism (χ^2^ = 25.88, *df* = *2, p* < 0.001). Overall 26.3% of participants reported “Action” (i.e., doing it at least once in the past school year), but 56.7% of those who judged it to be a “personal choice” and only 12.7% of those who judged it “morally wrong.” The same effect was also large for letting another student copying from one's own test (χ^2^ = 22.19, *df* = *2, p* < 0.001) and smaller but still significant for unpermitted collaboration (χ^2^ = 9.85, *df* = *2, p* < 0.01). The pattern of percentages was the same for the remaining three cheating behaviors, but the pattern of significant between-group effects varied. There were no differences for the first action (copying someone's else homework) and while only participants who judged the remaining two test cheating actions as a personal choice were significantly over-represented in reporting the action.

### Full model tests of direct and indirect effects of judgment on dishonesty

**Figure 3:**
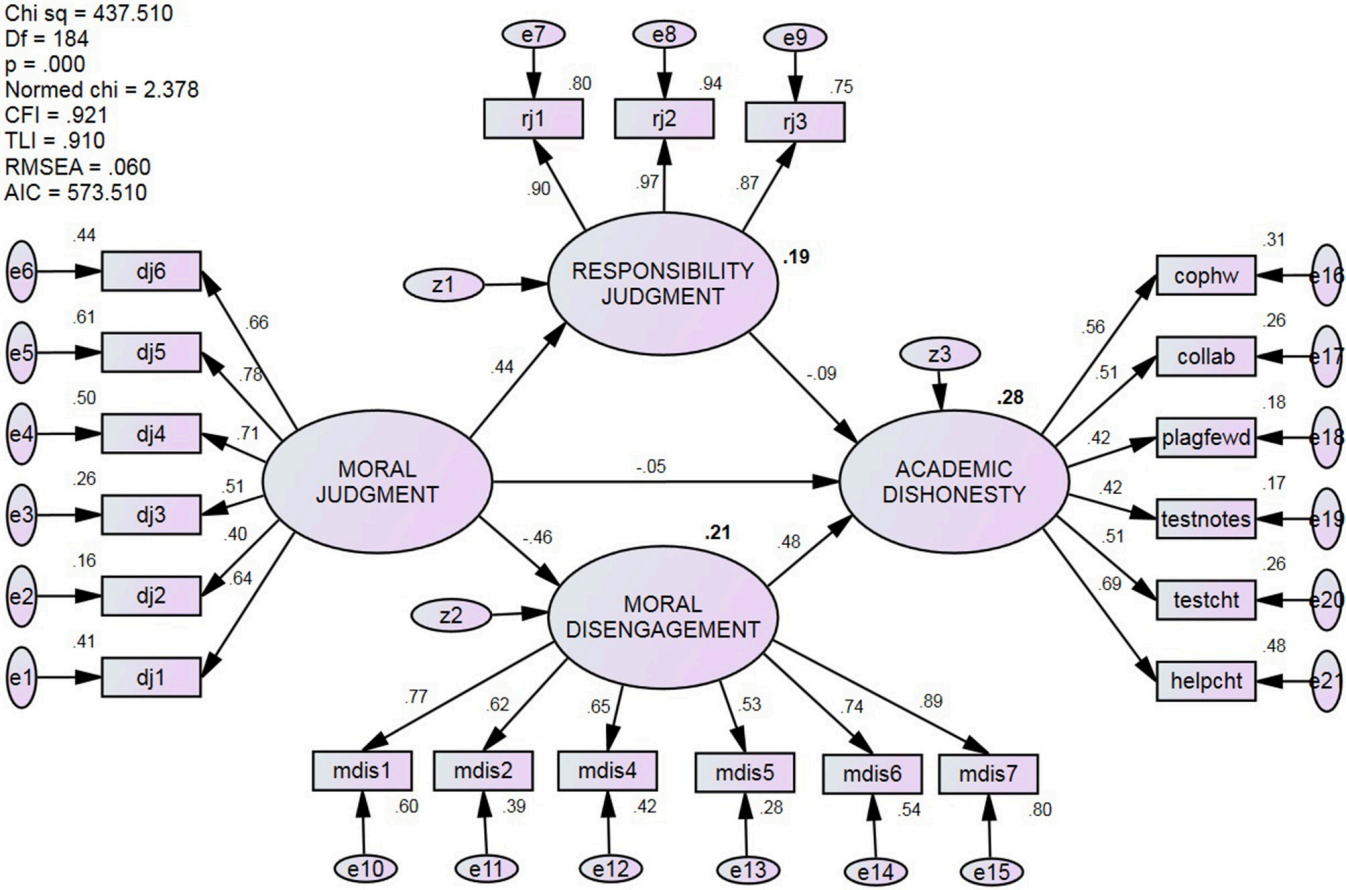
Standardized estimates and fit statistics of the hypothesized model. The model fit is acceptable, but did not fully support as the hypothesized model—neither the direct path from Moral Judgment, nor the indirect path through Responsibility Judgment, to Academic Dishonesty was significant.

As depicted in Figure 3, the data collected offered an acceptable fit to the hypothesized model: χ^2^/*df* = 2.38, CFI = 0.921, TLI = 0.910, Gamma hat = 0.941, RMSEA = 0.060 (90% CI = 0.053, 068), AIC = 573.51, and *r*^2^ = 0.28. However, several of the hypothesized effects were not supported. Specifically, moral judgment was expected to have both direct and indirect (through both responsibility judgment and moral disengagement) associations with academic dishonesty, but only showed an indirect effect through moral disengagement. Moral judgment was negatively predicted moral disengagement (β = −0.46, *p* < 0.001), which positively predicted academic dishonesty (β = 0.48, *p* < 0.001). The direct path from moral judgment to academic dishonesty was not significant (β = −0.05), nor was the indirect path from responsibility judgment to academic dishonesty (β = −0.09).

**Figure 4:**
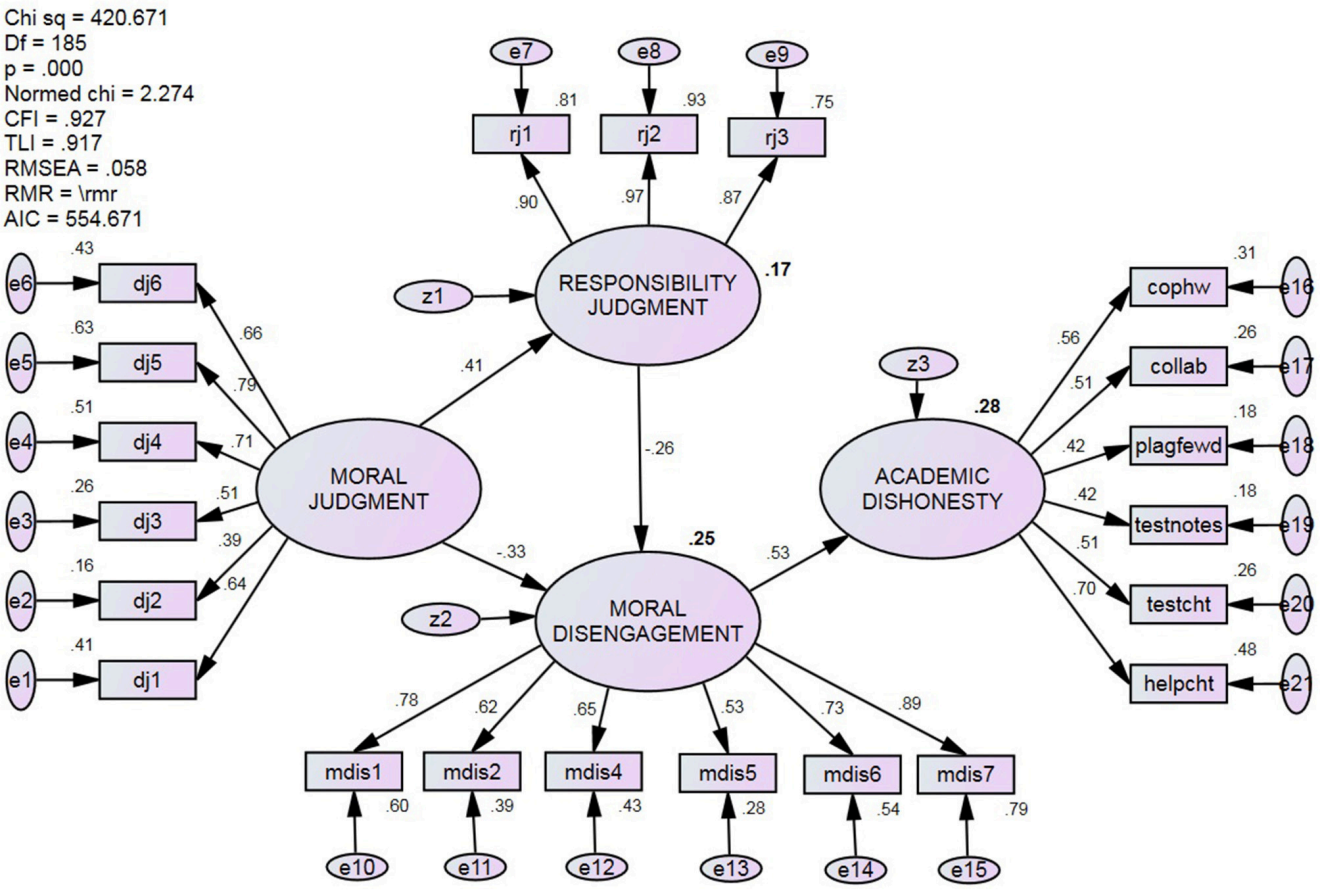
Standardized estimates and fit statistics of the modified model. Indirect effects of responsibility judgment on the link between moral judgment and moral disengagement, and moral disengagement on the link between moral judgment and cheating behavior. All paths significant, *p* < 0.001.

In the interest of retaining responsibility judgment in the model, and in light of its significant bivariate relations with all other factors (see Table 2), an exploratory SEM was conducted on a modified version of the hypothesized model. Specifically, as shown in Figure 4, the direct path from moral judgment to academic dishonesty was removed and the indirect path from responsibility to academic dishonesty was redirected to moral disengagement. The data collected offered an acceptable fit to the modified model: χ^2^/*df* = 2.27, CFI = 0.927, TLI = 0.917, Gamma hat = 0.944, RMSEA = 0.058 (90% CI = 0.051, 065), AIC = 554.67, and *r*^2^ = 0.28. As illustrated, the relations between moral judgment and moral disengagement were partially mediated by responsibility judgment, and moral disengagement fully mediated the relationship between moral judgment and academic dishonesty. The modified model explained the same amount of variance as the hypothesized model, but based on the difference in chi-square values, the data offered a better fit to the modified model (Δ in χ^2^ = 437.51_HYP_ − 420.67_MOD_ = 16.84, Δ in *df* 's = 185_HYP_ − 184_MOD_ = 1, *p* of Δ < 0.001).

Finally, the hypothesized model was expected not only to demonstrate a good fit, but offer a significantly better one than three less complex models (H5). As reported above, while data collected demonstrated an acceptable fit to the hypothesized model, a modified model demonstrated a better fit. Accordingly, both the hypothesized and modified models were tested against the three other models. As detailed in Table 4, the data demonstrated acceptable or good fit to all five of the models tested. However, the effect sizes of the models varied widely. Model 1 (the simplest model testing the direct effects of moral judgment on academic dishonesty) and Model 2 (with responsibility judgment as a mediator in the relations between judgment and dishonesty) explained relatively little variance (*r*^2^ = 0.08 and 0.11, respectively) compared to Model 3 (with moral disengagement as a mediator in the relations between judgment and dishonest) which explained the same amount of variance (*r*^2^ = 0.28) as Model 4 (hypothesized) and Model 5 (modified). In order to further compare Model 3 and 5, the observed changes in chi-square values were examined. Although Model 3 was more parsimonious, the data offered a better fit to the Model 5 (Δ in χ^2^ = 420.67_M5_ − 311.98_M3_ = 108.69, Δ in *df* 's = 185_M5_ − 132_M3_ = 53, *p* of Δ < 0.001).

**Table 4:** Full model fit statistics for five competing models of moral functioning.

**Model**	**χ^2^**	***df***	**χ^2^/*df***	**CFI**	**TLI**	**Gamma**	**RMSEA**	**CI of RMSEA**	**AIC**	***R*^2^**
M1: MJ - AD	141.95	53	2.68	0.901	0.885	0.962	0.067	(0.053−0.080)	215.95	0.08
M2: MJ - RJ - AD	211.50	87	2.43	0.940	0.927	0.959	0.061	(0.051−0.072)	307.50	0.11
M3: MJ - MD - AD	311.98	132	2.36	0.913	0.900	0.950	0.060	(0.051−0.069)	425.98	0.28
M4: MM Hypothesized	437.51	184	2.38	0.921	0.910	0.941	0.060	(0.053−0.068)	573.51	0.28
M5: MM Modified	420.67	185	2.27	0.927	0.917	0.944	0.058	(0.051−0.065)	554.67	0.28

*CI, 90% Confidence Interval; MJ, Moral Judgment; AD, Academic Dishonesty; RJ, Responsibility Judgment; and MD, Moral Disengagement; MM , Multi-mediation Model*.

## Discussion

The present investigation was guided by three purposes aimed at measuring moral judgment and its relations to academic dishonesty. Each purpose was itself guided by a set of research questions and hypotheses. Overall, as summarized in Table 5, results offered partial support for the hypotheses. The findings are discussed in further detail below.

**Table 5:** Summary of study purposes, hypotheses, and findings.

**Purposes and Hypotheses**		**Findings**
1st Purpose: To Assess Judgment Related to Academic Dishonesty		Academic Dishonesty is Wrong, and
H1	The majority of participants would judge academic dishonesty to be a matter of convention, with fewer students judging the behaviors assessed as morally wrong, and fewer still as a personal choice.	Partially supported—As predicted, the majority of participants judged unpermitted collaboration and plagiarism to be matters of convention; however, and unexpectedly, copying homework and the three test cheating items were judged morally wrong by the majority.
H2	Participants would be more likely to judge assignment cheating items as personal choices or conventional issues, and more likely categorize test cheating as morally wrong.	Partially supported—As predicted, participants were more likely to judge the three test cheating actions as morally wrong participants and unpermitted collaboration as a conventional violation; however, the difference was not significant for copying homework, as an unexpected majority of participants judged it to be morally wrong.
2nd Purpose: To Explore Judgment-Action Associations		Moral Judgment Matters, but
H3	Participants who judge behaviors to be morally wrong would report lower rates of engagement in that behavior compared to students who judged the behavior to be a personal choice or social convention.	Partially supported—As predicted, participants who judged an action morally wrong were less likely to report doing it compared to students who judged it a personal choice or social convention; however, the differences were statistically significant for only three of the six actions (i.e., unpermitted collaboration, plagiarism, and letting another copy from your test/exam).
3rd Purpose: To Test Competing Models of Moral Functioning		Moral Functioning is Multi-component and Effortful
H4	Moral judgment (MJ) would have a direct negative relationship with academic dishonesty (AD) as well as indirect associations through both responsibility judgment (RJ) and moral disengagement (MD).	Partially supported—As predicted (see Figure 1), MJ was indirectly related to academic dishonesty through MD; however, there was no direct effect of MJ on AD or an indirect effect on this relationship through RJ (see Figure 3). In light of the findings and theoretical considerations, the hypothesized model was modified by removing the direct effect of MJ on AD and repositioning RJ as a mediator between MJ and MD. The data offered a good fit to the modified model with all paths significant (see Figure 4).
H5	The hypothesized model was expected to demonstrate a good fit, and a significantly better one than any of three competing models.	Supported—As predicted, the data demonstrated an acceptable fit to the hypothesized model, but two of the hypothesized paths were not significant and a modified model was created. Both of these multiple mediation models were tested against the three competing models and against each other. The former two models were significantly better than the latter three in both goodness of fit and variance explained. A final test proved the data fit the modified model better than the hypothesized model.

The first purpose of this study was to assess students' judgments related to academic dishonesty and to do so with a new instrument based on social domain theory. This instrument asked participants to classify six examples academic dishonesty, and H1 posited that most participants would judge the actions assessed to be matters of *social convention* (e.g., “Wrong because it's against societal laws, rules or customs”) rather than morally wrong (“Wrong regardless of laws or rules”). In fact, with only two exceptions, the majority of participants the behaviors listed to be *morally wrong*. As expected, very few participants judged the behaviors to be a *personal choice*. Similarly, support for H2 was also mixed. As expected, compared to unpermitted collaboration, more participants judged the three test cheating behaviors to be *morally wrong*; however, and unexpectedly, most participants also judged the copying of homework to be *morally wrong*. In short, the vast majority (approximately 84–98%) of participants believed all six behaviors to be wrong—either as a violation of rules or moral principle—and test or exam cheating more as morally wrong.

The second purpose of the present study was to explore the associations between students' judgments and actions related to academic dishonesty. H3 predicted that students' who judged behaviors to be morally wrong reported significantly lower rates of engagement in that behavior compared to students who judged the behavior to be a personal choice, but not social convention (as also predicted). However, though not statistically significant, those who judged the action to be morally wrong reported the lower rates of engagement in all types of academic dishonesty. This pattern of findings comports with the previous research which has shown significant negative associations between cheating and the extent to which students believe it to be wrong, unacceptable, or unjustifiable (e.g., Anderman et al., 1998; Jordan, 2001; Murdock et al., 2004; Stephens et al., 2007).

**Figure 5:**
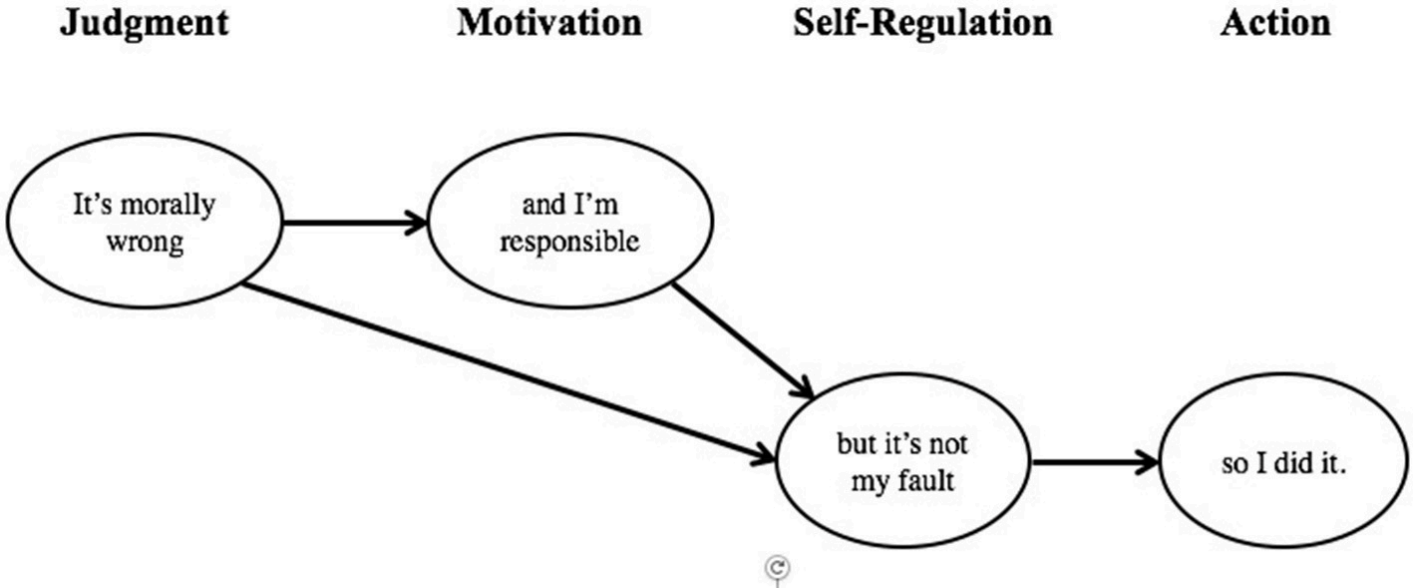
A multi-component conceptual model of moral functioning in the domain of academic dishonesty. Judgment (“It's morally wrong”) and motivation (“I'm responsible”) serve as protective factors against action (“I did it”), but can be undermined by selective deactivation of self-regulatory functions through mechanisms of moral disengagement (“It's not my fault”).

The third and final purpose of this study was to test several prominent theoretical models of moral functioning, and offered a hypothesized four-component model that positioned moral motivation and self-regulation as mediators of the relations between moral judgment and academic dishonesty. Although the hypothesized model demonstrated acceptable fit, it only offered partial support for H4—the direct path from moral judgment to academic dishonesty was not significant, nor its indirect path through responsibility judgment. A modified version of the hypothesized model was tested and demonstrated a better fit than the hypothesized model. This modified model indicated that self-regulation (in the form of selective *moral disengagement*) played a significant mediating role in the relations between moral judgment and academic dishonesty, and that moral motivation (i.e., *responsibility judgments*) meditated the relationship between moral judgment and moral disengagement. The relationships are summarized in Figure 5 as a narrative chain of events, whereby both moral judgment (“It's morally wrong”) and motivation (“I'm responsible”)—and the protective impetus and drive they offer against cheating—are neutralized through moral disengagement (“It's not my fault”) and result in a gap between judgment and action (“I did it”).

Finally, and consistent with H5, both the hypothesized and modified models demonstrated a better fit than the three more parsimonious models of moral functioning tested. The latter models included a simple bivariate model of the direct effects of moral judgment on academic dishonesty (as Socrates would have it), a tri-variate model with responsibility judgment mediating the relations between judgment and dishonesty (consistent with Kohlberg and Candee, 1984), and another tri-variate model with moral disengagement as the mediator (as suggested by Bandura, 1986, 1990). In short, findings from the present study suggest that while moral judgment is important, it is only one of several components needed for effective moral functioning; motivation and self-regulation play critical mediating roles in helping to bridge the divide between judgment and action.

## Significance of findings

The findings of the present investigation are significant in at least three important ways. Firstly, the findings associated the first purpose contribute to the literature on moral judgment related to academic dishonesty and its assessment. While most previous studies have measured *how much* they believed academic dishonesty to be “wrong,” “serious,” or “unacceptable” (e.g., Anderman et al., 1998; Jordan, 2001; Murdock et al., 2004; Stephens et al., 2007), the present study developed and used a new instrument rooted in SDT (Turiel, 1983; Nucci, 2001; Smetana, 2006) that assessed *what domain* of behavior students use to classify or judge academic dishonesty. In doing so, the present investigation demonstrated that judgments of cheating behavior vary not only in degree (as typically assessed) but also in kind (as social domains). The domain distinctions made—personal, conventional, or moral—have important implications for moral functioning (as discussed next), and the instrument presented here offers researchers a valid and reliable approach to investigating these distinctions as they relate to academic dishonesty.

Findings associated with this study's second purpose—the associations between domain judgments and academic dishonesty—are of particular significance for educators well as those developing courses or programs aimed at promoting academic integrity. Specifically, the results clearly indicate that moral judgment matters (students who deemed an action “morally wrong” where less likely to report doing it) and that not all students see academic dishonesty (in its various forms) as morally wrong. Taken together these findings suggest that education is both warranted and needed. Specifically, teachers and developers should look for opportunities to engage students in discussions about academic integrity—what it means, why it's important, and especially how academic dishonesty (in all its forms) involves moral issues and values (e.g., fairness, honesty, and trust). Fortunately, the rising tide of concern over academic cheating over the past decade has produced several approaches for promoting academic integrity, including classroom-based seminars for secondary students (e.g., Stephens and Wangaard, 2016) as well as school-wide interventions aimed at “cultivating integrity” (e.g., Seider et al., 2013; Stephens and Wangaard, 2013).

The final contribution of this study concerns the findings related to its third purpose involving the testing of several theoretical models of moral functioning. These findings (even if modestly) contribute to the long-standing and on-going dialog among psychologists, philosophers, and others interested in the judgment-action gap or moral functioning more broadly. In particular, results from the present study add to the existing literature by offering four-component model of moral functioning that helps bridge the divide between judgment and action as it relates to academic dishonesty. While such multiple-component models are not unique to the literature (e.g., Kohlberg and Candee, 1984; Rest, 1986; Beck and Ajzen, 1991; Rest et al., 1999; Olafson et al., 2012), the final model supported in this study brings together important constructs from several theoretical perspectives in a novel way that highlight the role of motivational and self-regulatory factors in bridging the divide between belief and behavior. Importantly, the results illustrate that while moral judgment is necessary for moral functioning, it is only one among many components needed for effective moral functioning—motivation derived from a sense of the personal responsibility and the activation of self-regulatory subfunctions (and not their disengagement) are needed as well if a judgment-action gap is to be mitigated.

Finally, findings from the present investigation have important implications of educators and policy makers interested in reducing the widespread problem of academic dishonesty. Specifically, these findings suggest that intervention efforts should not only focus on strengthening students' moral judgments related to cheating, but also on forming both a sense of personal responsibility for not cheating *and* a resistance (what Kohlberg called “ego strength”) to undermining that sense of responsibility through moral disengagement. In short, more holistic approaches are needed to ameliorate the widespread problem of academic dishonesty (Stephens, 2016).

## Limitations and future directions

The present study used a cross-sectional research design, where data was collected at a single point of time via an anonymous self-report survey. While convenient and cost-effective, this design and method of data gathering presents limits with respect to making firm causal claims (Campbell and Fiske, 1959). Structural equation modeling was used to simulate potential causal effects, but more robust research designs are needed to affirm of relations found in the present study. Future studies should employ longitudinal or experimental designs to assess the causal effects of moral judgments on academic dishonest and mediating roles of motivational and self-regulatory factors. Another important limitation concerns the generalizability of the findings. The sample of participants were from a single source—a relatively small one of secondary students at a highly selective private school in the northeastern United States (Podsakoff and Organ, 1986). Future research should seek to procure larger, more demographically-diverse samples to assess the validity of the model (or ones like it) in different contexts and populations.

Finally, the present study was limited with respect to the processes and outcomes selected for investigation. While there were sound theoretical and methodological reasons for the four constructs included (and results confirmed their validity and importance), student engagement in academic dishonesty is must certainly a function of many factors beyond the four assessed here. Future research should include of other social-cognitive processes as well as situational and cultural variables. The models tested here were limited to the former processes, and did well to explain 28% of variance in academic dishonesty, but much remains to be studied and understood in bridging the divide between students' beliefs and behaviors related to cheating.

## Conclusion

The results of the present study lead to following flow of conclusions: (1) most students' believe academic dishonesty is wrong—conventionally or morally—but judgments vary across individuals and types of cheating behavior more educational opportunities are needed to help students perceive and reason through the moral dimensions of cheating, because; (2) moral judgment matters—students who believe an action to be morally wrong are less likely to engage in it, however; (3) moral functioning is multi-component and effortful—while moral judgment may be necessary for moral action, its relationship is best understood as indirect, requiring the support of motivational variables (such as a sense of personal responsibility for not cheating) and one's related to self-regulatory ones (such as mechanisms of moral disengagement that counter the influences of moral judgment and motivation) to bridge the oft-observed divide between them.

## Author contributions

As the sole author, JS was responsible for the conception of the work as well as all analysis and interpretation of data, the creation of all tables and figures, and all writing associated with the manuscript.

### Conflict of interest statement

The author declares that the research was conducted in the absence of any commercial or financial relationships that could be construed as a potential conflict of interest.
